# Allopolyploid Origin of *Chenopodium album* s. str. (Chenopodiaceae): A Molecular and Cytogenetic Insight

**DOI:** 10.1371/journal.pone.0161063

**Published:** 2016-08-11

**Authors:** Karol Krak, Petr Vít, Alexander Belyayev, Jan Douda, Lucia Hreusová, Bohumil Mandák

**Affiliations:** 1 Institute of Botany, Czech Academy of Sciences, Zámek 1, CZ-252 43, Průhonice, Czech Republic; 2 Faculty of Environmental Sciences, Czech University of Life Sciences Prague, Kamýcká 129, CZ-165 21, Praha 6 – Suchdol, Czech Republic; Georg-August-Universitat Gottingen, GERMANY

## Abstract

Reticulate evolution is characterized by occasional hybridization between two species, creating a network of closely related taxa below and at the species level. In the present research, we aimed to verify the hypothesis of the allopolyploid origin of hexaploid *C*. *album* s. str., identify its putative parents and estimate the frequency of allopolyploidization events. We sampled 122 individuals of the *C*. *album* aggregate, covering most of its distribution range in Eurasia. Our samples included putative progenitors of *C*. *album* s. str. of both ploidy levels, i.e. diploids (*C*. *ficifolium*, *C*. *suecicum*) and tetraploids (*C*. *striatiforme*, *C*. *strictum*). To fulfil these objectives, we analysed sequence variation in the nrDNA ITS region and the *rpl*32-*trn*L intergenic spacer of cpDNA and performed genomic *in-situ* hybridization (GISH). Our study confirms the allohexaploid origin of *C*. *album* s. str. Analysis of cpDNA revealed tetraploids as the maternal species. In most accessions of hexaploid *C*. *album* s. str., ITS sequences were completely or nearly completely homogenized towards the tetraploid maternal ribotype; a tetraploid species therefore served as one genome donor. GISH revealed a strong hybridization signal on the same eighteen chromosomes of *C*. *album* s. str. with both diploid species *C*. *ficifolium* and *C*. *suecicum*. The second genome donor was therefore a diploid species. Moreover, some individuals with completely unhomogenized ITS sequences were found. Thus, hexaploid individuals of *C*. *album* s. str. with ITS sequences homogenized to different degrees may represent hybrids of different ages. This proves the existence of at least two different allopolyploid lineages, indicating a polyphyletic origin of *C*. *album* s. str.

## Introduction

How new forms arise in nature is a key question in evolutionary biology. It is believed that a significant part of speciation and diversification in plants (particularly in angiosperms and ferns) involved reticulate evolution characterized by occasional hybridization creating a network of closely related taxa below and at the species level [1]. A new hybridogenous species may have the same number of chromosomes as its parents (homoploid hybridization), or be a descendant of either (i) cross-fertile progenitors that underwent doubling of structurally homologous genomes, i.e. autopolyploidy, or (ii) cross-sterile individuals escaping from sterility by chromosome doubling, i.e. allopolyploidy [2–7]. Consequently, hybridization can lead to rapid genomic changes, including chromosomal rearrangements, genome expansion, differential gene expression and gene silencing [8, 9]. These genomic changes may lead to beneficial new phenotypes, and selection for ecological traits may in turn alter genome structure [10]. Besides, interspecific hybridization introduces evolutionary novelties by combining the parental species’ traits. Due to the contribution of previously isolated (and therefore diverse) genotypes, hybrids are supposed to have higher genetic diversity compared to their parents [11–14].

The increasing attention of biologists to polyploidy in the last few decades went hand-in-hand with the development of new karyological, cytogenetic and molecular methods enabling the study of mechanisms, patterns and processes behind polyploid formation. One of the major achievements of these approaches is the finding that polyploid species may have been formed multiple times, independently (i.e. polyphyletically), as a result of hybridization between genetically divergent parental species [15]. An increasing number of case studies has since confirmed that allopolyploids are very frequently of polyphyletic origin and that this should be considered the rule rather than the exception, e.g. [16–21].

The genus *Chenopodium* sensu lato has a worldwide distribution with the highest species diversity in temperate areas; it includes at least 150 species [22, 23]. Karyological studies of the genus are relatively abundant [24–35]; see [7] for summary of chromosome counts for European *C*. *album* agg., and indicate the presence of two basic chromosome numbers (x = 8, 9) and different ploidy levels (diploid, tetraploid, hexaploid and decaploid). The common occurrence of polyploidy within the *C*. *album* aggregate is, however, not satisfactory explained, and we have only fragmentary information about the origins of individual polyploid species. For example, two parental subgenomes participated in the origin of the genome of the South American tetraploid species *C*. *quinoa* Willd. While one of the parents was related to North American *C*. *standleyanum* Aellen/*C*. *incanum* (S. Watson) Heller or another closely related diploid, the other parent was close to Eurasian *C*. *suecicum* J. Murr/*C*. *ficifolium* Sm. or another related diploid species [36, 37]. The economically exceptionally important tetraploid *C*. *quinoa* is an allopolyploid, but there are many other polyploids within the *C*. *album* group with completely unknown history, including one of the world’s worst weeds *C*. *album* s. str. The *C*. *album* aggregate is one of the taxonomically most challenging groups in the genus. It comprises morphologically highly variable taxa, resulting in the description of many hybrids [38–42] and hundreds of microspecies or intraspecific entities [43]. However, according to some authors [22, 44, 45], only a few basic, morphologically very variable, species should be recognized. The large morphological variation, especially of *C*. *album* L. s. str., should instead be explained by wide phenotypic plasticity, common interspecific hybridization between species with different ploidy levels (refuted by Mandák et al. [7]) or by the presence of various ploidy levels within species. However, Mandák et al. [7] has shown that individual species are very tightly correlated with some specific ploidy level (see also [46]). They showed that, at least in Europe, the *C*. *album* aggregate comprises the following six widely distributed species (excluding extremely rare or adventive species) with the following ploidies: diploids (2n = 2x = 18) *C*. *suecicum* and *C*. *ficifolium*, tetraploids (2n = 4x = 36) *C*. *strictum* Roth and *C*. *striatiforme* J. Murr, and hexaploids (2n = 6x = 54) *C*. *album* s. str. and *C*. *opulifolium* W. D. J. Koch et Ziz. Hence, the wide morphological variation detected, especially in polyploids, might be a consequence of their allopolyploid origin, caused by a combination of multiple genomes hypothetically participating in the polyphyletic evolution of this widely distributed species. To test this, we need to know the origin of each polyploid species.

Recently, several attempts were made to elucidate the origin of the hexaploid *C*. *album* s. str. [7, 37, 47, 48]. However, neither of them has brought a satisfactory explanation. Gangopadhyay et al. [47] speculate about the involvement of three diploid species. According to them, narrow-leaved and broad-leaved diploid cytotypes of *C*. *album* and *C*. *murale* L., a species not belonging to the *C*. *album* aggregate, were involved. According to Rahiminejad & Gornall [48] the diploids *C*. *ficifolium* and *C*. *suecicum* (or taxa very similar to these two) are considered the putative parents of *C*. *album* s. str., based on an analysis of secondary metabolites. Mandák et al. [7] compared four hypothetical scenarios, including that of Rahiminejad & Gornall [48], by analysing genome size variation among European taxa belonging to the *C*. *album* aggregate. Hybridization between diploid and tetraploid taxa via unreduced gametes turned out to be the most likely scenario. Nonetheless, genome size was unable to discriminate between species with the same ploidy level, so the exact combination of parental taxa of *C*. *album* s. str. remains unclear. The allopolyploid origin of *C*. *album* s. str. was recently confirmed by the molecular systematic study of Walsh et al. [37]. No study, however, was able to identify the putative parents of *C*. *album* s. str. The study of Mandák et al. [7] thus still represents the closest approximation of the species origin.

Fuentes-Bazan et al. [49, 50] sequenced the nrDNA ITS region, but did not report on the occurrence of sequence heterogeneity in *C*. *album* s. str. However, our pilot study revealed a certain degree of intraindividual polymorphism in ITS sequences of this species, indicating that this marker may be a valuable source of information about its origin. We therefore sampled *C*. *album* s. str. accessions covering a significant part of the species’ distribution range in Eurasia (from the Iberian Peninsula to western China). Besides the analysis of ITS, we sequenced cpDNA and performed genomic in situ hybridization (GISH), aiming to: (1) verify the hypothesis of Mandák et al. [7] that *C*. *album* s. str. originated via hybridization between diploid and tetraploid species; (2) identify the direction of the hybridization; (3) evaluate the pattern of intraspecific variation of *C*. *album* s. str. at the continental and local scales; and (4) estimate whether *C*. *album* s. str. has a mono- or polyphyletic origin.

## Materials and Methods

### Ethics statement

The collections used for this study did not involve endangered or protected species, and no specific permissions were required for sampling activities in these locations.

### Plant material

We sampled 52 populations of hexaploid *Chenopodium album* s. str., each represented by 1 accession, covering most of the species distribution range in Eurasia. In addition to this coarse sampling, we sampled 5–13 individuals from five populations in the Czech Republic to investigate genetic variation on a fine scale. In total, we sampled 96 individuals of *C*. *album*. Further, we sampled potential parental species of *C*. *album*: diploid *C*. *ficifolium* (5 accessions) and *C*. *suecicum* (9 accessions) as well as tetraploid *C*. *striatiforme* (2 accessions) and *C*. *strictum* (8 accessions). *Chenopodium chenopodioides* (L.) Aellen, *C*. *glaucum* L., *C*. *rubrum* L., and *C*. *urbicum* L. served as outgroup taxa. The origins of plant material used for the present study are given in Table 1. When available, ripe seeds were collected in the field; if not, leaf material from well-developed flowering plants was collected and dried in silica gel and stored until DNA extraction. The plants were grown in the experimental garden of the Institute of Botany, Czech Academy of Sciences, Průhonice, Czech Republic (49.991667, 14.566667, *ca*. 320 m above sea level) between 2011 and 2015. The seeds were germinated in 5 × 5 cm bedding cells with homogenous garden compost and later moved to 19 × 19 × 19 cm (6.9 L) pots filled with common garden substrate. Fresh leaves were collected from each accession and stored in silica gel until DNA extraction. For genomic in situ hybridization, seeds from selected accessions were germinated again and seedlings were cultivated. Root tips were sampled from young plants and stored in a fixative solution (3:1 mixture of absolute ethanol and acetic acid) prior to the analyses.

**Table 1:** Material used in the present study.

*Species*	Locality/Specimen no.	Origin	Latitude	Longitude	cpDNA	ITS	Note
*Chenopodium album*	64/ PRA-11130	Czech Republic, Praha-Holešovice	50.112559	14.444746	3 (KU517367)	7	
	144/ PRA-11131	Hungary, Rajka	48.010200	17.176600	1	7	
	165/ PRA-11151	Bulgaria, Opletnya	43.089983	23.466000	1	7	
	216	Croatia, Brac Island, Bol	43.261342	16.658225	1	7 (KU517382)	
	235/ PRA-11132	Denmark, Falster Island, Gedser Remise	54.575275	11.925328	1	7	
	329/ PRA-11135	Czech Republic, Slatina	50.226388	14.210527	1	7	GISH
	347/ PRA-11157	Romania, Constanta	44.719750	25.137361	1	7	
	358/ PRA-11158	Bulgaria, Asenovgrad	42.023639	24.859278	1	7	
	369	Albania, Fushë-Krujë	41.499056	19.699000	1	7	
	370/ PRA-11161	Albania, Rec	42.237972	19.527861	1	7	
	372/ PRA-11162	Croatia, Podgradina	43.008972	17.550694	1	7	
	402/ PRA-11165	China, Xinjiang, Tian Shan, Xibaiyang Goucun	43.449528	87.198306	1	7	
	423/ PRA-11166	China, Xinjiang, Altai, Kanas	48.675917	87.026722	1	7	
	439/ PRA-11167	China, Xinjiang, Hoxud	42.291917	86.860417	1	7	
	457/PRA-11136	China, Xinjiang, Tumuxiukezhen	41.534139	79.753778	1	7	GISH
	477/ PRA-11137	Russian Federation, Tuva, Ak-Chyraja	50.700000	93.260000	1	7	
	503/ PRA-11139	Russian Federation, Krasnojarsk area, Western Sajan	56.560000	91.290000	1	7	
	510/ PRA-11168	Russian Federation, Tuva	51.166667	93.566667	1	7	
	512/ PRA-11141	Russian Federation, Altai Republic, Barnaul	53.250000	83.683333	1	7	
	520/ PRA-11169	Great Britain, Huddersfield	53.642462	–1.791060	1	7	
	606/ PRA-11143	France, Chateauneuf	45.562850	–0.012100	1	7	
	609/ PRA-11144	Spain, Deba	43.296510	–2.356660	1	7	
	610/ PRA-11145	Spain, Deba	43.296510	–2.356660	1	7	
	618	Spain, Torrão do Lameiro	40.832570	–8.664490	1	7	
	621/ PRA-11146	Portugal, Torreira	40.764400	–8.700631	1 (KU517365)	7	
	624/ PRA-11173	Portugal, Celorico da Beira	40.627550	–7.432720	2 (KU517366)	7	
	634/ PRA-11147	Spain, Alagón	41.789960	–1.135400	1	7	
	502/ PRA-11138	Russian Federation, Kalmykia	46.300000	44.283333	1	7	
	608/ PRA-11170	France, Mimizan-Plage	44.211887	–1.293935	1	7	
	620/ PRA-11172	Portugal, Torreira	40.764400	–8.700631	1	7	
	631/ PRA-11176	Spain, Torrellas	41.899690	–1.768740	2	7	
	632/ PRA-11177	Spain, Alagón	41.764720	–1.125120	1	7	
	142	Slovakia, Nižné Čabiny	49.176172	21.908428	1	8	
	163/ PRA-11150	Bulgaria, Veliko Tarnovo	43.060583	25.632583	1	8	
	303/ PRA-11134	Slovakia, Nána	47.806944	18.695444	1	8 (KU517383)	
	339/ PRA-1155	Romania, Andrei Saguna	46.293944	21.392500	1	8	
	604/ PRA-11142	France, Étampes	48.445120	2.116930	1	8	
	157/ PRA-11148	Romania, Preluci	46.460967	26.267833	1	9 (KU517384)	
	160/ PRA-11149	Romania, Mihai Viteazu	44.673530	28.684520	1	10 (KU517385)	
	169/ PRA-11152	Bosnia and Herzegovina, Maglaj	44.555100	18.106283	1	11 (KU517386)	
	291/ PRA-11153	Czech Republic, Hrádek	48.781583	16.261528	1	12 (KU517387)	GISH
	299/ PRA-11133	Hungary, Fülöpháza	46.881944	19.455694	1	13 (KU517388)	
	333/ PRA-11154	Hungary, Hortobágy	47.581250	21.158417	1	13	
	343/ PRA-11156	Romania, Pitesti	44.867000	24.892889	1	14 (KU517389)	
	364/ PRA-11159	Bulgaria, Vranja	41.447222	23.404833	1	15 (KU517390)	
	507/ PRA-11140	Russian Federation, Novosibirsk	54.818050	83.143134	1	15	
	366/ PRA-11160	Greece, Ioannina	39.70000	20.813250	1	16 (KU517391)	
	612/ PRA-11171	Spain, Sahagún	42.369261	–5.019539	1	16	
	626/ PRA-11174	Spain, Salamanca	40.954080	–5.640250	1	16	
	374/ PRA-11163	Croatia, Saborsko	44.981806	15.476361	1	17 (KU517392)	
	375/ PRA-11164	Slovenia, Gabrje	46.198194	13.692722	1	18 (KU517393)	
	628/ PRA-11175	Spain, Villacastín	40.795520	–4.378170	1	19 (KU517394)	
	583/ PRA-11205, 11206, 11207	Czech Republic, Hostivice	50.075298	14.267106	1	7, 8, 9, 20 (KU517395), 21 (KU517396)	Population data, 10 individuals sampled
	565/ PRA-11202	Czech Republic, Praha-Bubeneč	50.112500	14.412583	1	7, 8, 13, 22 (KU517397)	Population data, 13 individuals sampled
	568/ PRA-11203	Czech Republic, Podbořany	50.241944	13.423278	1	7, 8, 9, 21	Population data, 7 individuals sampled
	579/ PRA-11204	Czech Republic, Podbořanský Rohozec	50.204889	13.284833	1	7, 8,23 (KU517398)	Population data, 5 individuals sampled
	599/ PRA-11208, 11209, 11210, 11211	Czech Republic, Vrané nad Vltavou	49.941808	14.395150	1	7, 8, 15, 16, 24 (KU517399), 25 (KU517400), 26 (KU517401), 27 (KU517402)	Population data, 9 individuals sampled
*Chenopodium ficifolium*	276/ PRA-11181	Czech Republic, Nový Bydžov	50.234361	15.428778	7 (KU517371)	1 (KU517376)	GISH-probe
	307/ PRA-11178	Slovakia, Nána	47.806944	18.695444	7	1	
	458/PRA-11179	China, Xinjiang, Tumuxiukezhen	41.534139	79.753778	7	1	
	466/PRA-11180	China, Xinjiang, Urumqi	43.855833	87.569722	7	2 (KU517377)	
	559	Great Britain, Huddersfield	53.646534	–1.784848	7	3 (KU517378)	
*Chenopodium striatiforme*	267/ PRA-11183	Latvia, Strenči	57.619489	25.701168	1	14	
	331/ PRA-11184	Czech Republic, Mělník	50.349528	14.497444	1	7	GISH-probe
*Chenopodium strictum*	380/PRA-10917, 10918	Czech Republic, Praha-Troja	50.115964	14.433326	1	7	GISH-probe
	480/ PRA-11187	Russian Federation, Samara area, Zhigulevsk	53.416667	49.533333	1	7	
	166/ PRA-11188	Bulgaria, Yakoruda	42.006833	23.640083	4 (KU517368)	28 (KU517403)	
	309/ PRA-11185	Slovakia, Virt	47.743250	18.325889	1	28	
	336/ PRA-11189	Hungary, Hortobagy	47.581250	21.158417	1	28	
	456/PRA-11191	China, Xinjiang, Tumuxiukezhen	41.534139	79.753778	1	28	
	478/ PRA-11186	Russian Federation, Volgograd area	49.100000	46.119722	1	28	
	434/ PRA-11190	China, Xinjiang, Altai, Hoboksar	46.541472	85.358083	1	29 (KU517404)	
*Chenopodium suecicum*	156/ PRA-11199	Romania, Preluci	46.460967	26.267833	5	4 (KU517379)	
	213/ PRA-11193	Russian Federation, Novosibirsk	54.854694	83.193444	5	5	
	319/ PRA-11200	Russian Federation, Archangelsk area, Archangelsk	64.507966	40.658118	5	4	
	324/ PRA-11196	Russian Federation, Moscow area, Dolgoprudnyj	55.938200	37.473167	5	4	
	513/ PRA-11198	Russian Federation, Altai Republic, Barnaul	53.250000	83.683333	5	4	
	264//PRA-11194	Lithuania, Rumšiškes	54.879404	24.197950	5	5	
	277/PRA-11195	Czech Republic, Nový Bydžov	50.234361	15.428778	5	5 (KU517380)	GISH-probe
	328/ PRA-11197	Czech Republic, Švermov	50.176806	14.105472	5 (KU517369)	5	
	508	Russian Federation, Saratov area, Balakovo	51.933333	47.716667	6 (KU517370)	6 (KU517381)	
*Outgroup*							
*Chenopodium chenopodioides*	633/PRA-11213	Spain, Alagón	41.791680	–1.134290	KU517375	KU517405	
*Chenopodium rubrum*	465/PRA11212	China, Xinjiang, Toxun	42.800556	88.639222	KU517372	KU517406	
*Chenopodium urbicum*	337/PRA-11201	Romania, Ciumeghiu	47.579500	21.148194	KU517374	KU517407	
*Chenopodium glaucum*	272/PRA-11182	Czech Republic, Rohovládova Bělá	50.110556	15.604083	KU517373	KU517408	

Information on the origin, cpDNA haplotype (cpDNA) and nrDNA ITS ribotype (ITS) is presented for all accessions analyzed. The GenBank accessin numbers of the representative sequences for each cpDNA haplotype and nrDNA ribotype are given by the accession that was submitted to the database. Samples used for chromosome spread preparation for GISH experiments are marked with “GISH”, and samples used as probes for GISH experiments are marked with “GISH-probe”. The herbarium specimens are deposited in the herbarium of the Institute of Botany of the Czech Academy of Sciences (PRA).

### DNA extraction, PCR and sequencing

Total genomic DNA was extracted following the sorbitol extraction method [51]. The non-coding *rpl*32-*trn*L region of chloroplast DNA was analysed to determine the maternal lineage of *C*. *album* s. str. The primers described in [52] were used. The PCRs were carried out in 25 μl reactions containing 12.5 ul of Plain PP MasterMix (TopBio, Prague, Czech Republic), 0.2 μM of each primer and 20 ng of genomic DNA. The cycling conditions were as follows: 4 min at 95°C followed by 35 cycles of 95°C for 30 s, 55°C for 30 s and 72°C for 1 min, and final extension at 72°C for 15 min.

The nrDNA ITS region was amplified using the primers AC-ITS5 [49] and ITS4 [53]. The PCRs were carried out in 25 μl, each reaction containing 1 x PCR buffer with KCl (Fermentas, St. Leon-Rot, Germany), 3 mM of MgCl_2_, 0.2 mM of each dNTP, 0.2 μM of each primer, 0.5 U of Taq DNA polymerase (Fermentas) and 20 ng of genomic DNA. The cycling conditions were as follows: 4 min at 95°C followed by 35 cycles of 95°C for 30 s, 50°C for 30 s and 72°C for 1 min, and final extension at 72°C for 10 min. All samples were amplified in three independent reactions that were pooled equimolarly prior to any downstream analysis to minimize the stochastic effects leading to the elimination of underrepresented ITS sequence types during the amplification step.

The samples were purified and both strands were sequenced using the services of Macrogen (Amsterdam, The Netherlands).

One individual of hexaploid *C*. *album* s. str. showing a high level of intraindividual polymorphism in ITS was cloned to confirm the presence of ribotypes inferred based on direct sequencing. The TOPO TA cloning kit (Invitrogen, Carlsbad, CA) was used following the manufacturer's instructions, only downscaled to half reactions. Ten positive clones were transferred into 20 μl ddH and denatured at 95°C for 10 min. They served as templates for subsequent PCR amplifications and sequencing. Representative sequences were deposited in GenBank under the accession numbers KU517365– KU517408 (Table 1).

### Analysis of DNA sequences

Sequences were proofread and corrected manually for inadequate base calling using ChromasLite v 2.1 (Technelysium Pty., Brisbane, Queensland, Australia). The ITS sequences were checked for intraindividual polymorphism, and if mixed bases occurred at the same position in both strands, IUPAC ambiguity codes were used to mark these sites. Sequence alignments were done using the MAFFT algorithm [54] as implemented by the GUIDANCE web service [55]. Alignments were adjusted manually using BioEdit [56]. Mononucleotide repeats were excluded from the cpDNA dataset due to a potentially high level of homoplasy [57] prior to further processing of the data. The “simple gap coding” method [58], as implemented in SeqState [59], was used to score variation in indels.

### Phylogenetic analysis of cpDNA

Maximum parsimony (MP) analysis using heuristic search with 100 replicates of random sequence addition and TBR branch swapping, as implemented in PAUP* 4.0.b10 [60], was performed. Bayesian analysis (MB) was conducted using MrBayes 3.2.1 [61]. The GTR model was used as the best fitting model of nucleotide substitution as determined by the Hierarchical Likelihood Ratio Test, carried out using MrModeltest 2.3 [62]. Two parallel analyses with four chains each were run for 2 000 000 generations, sampling each 1000-th generation. The average deviation of split frequencies indicated that this number of generations was sufficient to reach convergence. Results of the first 500 steps were discarded as burn-in and the remaining 3002 trees were used to reconstruct the consensus tree. The MP and MB analyses were performed twice.

The haplotype network was reconstructed using TCS 1.21 [63]. A considerable part of the observed genetic variation was represented by insertions and deletions. However, TCS cannot work with the presence/absence matrix created for indels and appended to the sequence alignment file by SeqState. To include this information in the analysis, the 0/1 characters were replaced manually by A/T in the nexus input file. The analysis was then run with the default conditions (i.e. with gap characters treated as missing data and a 95% connection limit), so the software could analyse information on indels as A/T variation, but the original gap characters (represented by “-” in the sequence alignment) were not taken into account. This approach allowed the incorporation and equal weighting of indels with different lengths in the analysis.

### Phylogenetic analyses of ITS

Nucleotide diversities were calculated in MEGA 5.05 [64]. Maximum parsimony (MP) was performed as described for the cpDNA. The SYM+G model of nucleotide substitution was used for the MB analysis and the analysis was run for 1 000 000 generations, sampling each 1000-th generation. Results of the first 200 steps were discarded as burn-in and the remaining 1602 trees were used to reconstruct the consensus tree.

Neighbour net analysis as implemented in SplitsTree 4.12.6 [65], with ambiguous characters treated as average states, and uncorrected P distance was performed to better visualize the relationships of hexaploid ribotypes to those originating from diploids and tetraploids. Bootstrapping with 1000 replicates was performed to assess the support of the resulting groups.

### Chromosome preparations and genomic in situ hybridization (GISH) procedure

Root tips were fixed in 3:1 (v/v) 100% ethanol:acetic acid. The fixed root meristems were thoroughly washed in water and enzyme buffer (10 mM citrate buffer at pH 4.6), and partially digested in 0.3% (w/v) cytohelicase, pectolyase and 0.5% (w/v) cellulase (Sigma Aldrich, St. Louis, MO, USA) at 37°C for 2–3 hours followed by washes in enzyme buffer and water [66, 67]. The material, in a water drop, was carefully transferred onto a grease-free microscope slide and the cells were spread according to the technique described in [68] with modifications according to [69]. Slides were examined and metaphase chromosomes were photographed in phase-contrast under an Olympus BX-53 microscope.

For GISH experiments, total genomic DNA of *C*. *ficifolium*, *C*. *suecicum*, *C*. *strictum*, *C*. *striatiforme* and *C*. *album* s. str. were used. DNA was sonicated in a BIORUPTOR PICO (Diagenode, Liege, Belgium) machine and labelled with Cy3 (GE Healthcare Life Sciences, Little Chalfont, UK) and ATTO 488 (Jena Bioscience, Jena Germany) according to the standard oligolabelling protocol. Two differently labelled DNA probes were hybridized simultaneously and GISH was carried out on a ThermoBrite (StatSpin, Hannover, Germany) programmable temperature controlled slide processing system at 63°C for 3 h. Slides were stained with DAPI and mounted in antifade mountant (Vector Laboratories, Peterborough, UK). After the GISH procedure, the chromosomes were again examined and photographed under a phase contrast microscope and the resulting images were computerized using the ProgRes MF Cool system (Laboratory Imaging, Prague, Czech Republic).

## Results

### CpDNA haplotype diversity and phylogenetic relationships

**Fig 1:**
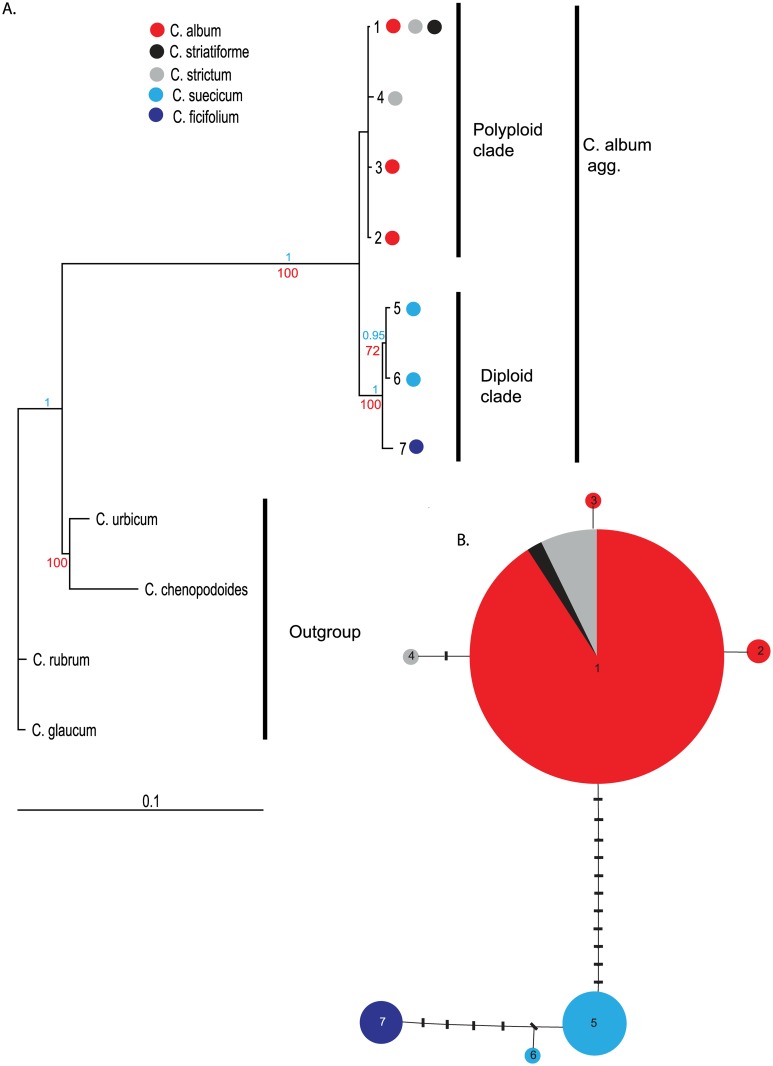
Analysis of cpDNA haplotypes. (A) A 50% majority rule consensus tree of the Bayesian analysis. The designated haplotype numbers are in accordance with Table 1; each haplotype is represented by one sequence. Numbers above and below branches indicate posterior probability (blue) and bootstrap support (red) values from Bayesian and maximum parsimony analysis, respectively. The presence of the particular haplotypes in species of the *Chenopodium album* agg. is indicated by coloured dots (see legend). (B) A cpDNA haplotype network. Each line represents one mutational step. Black bars represent missing haplotypes. The seven haplotypes identified in this study are represented by coloured circles. The size of each circle is proportional to the frequency of the particular haplotype. The occurrence of particular haplotypes in species of the *C*. *album* agg. is indicated by colours (see legend).

The total length of the *rpl*32-*trn*L alignment was 1254 bp including outgroup taxa (1184 bp when only the samples of *C*. *album* agg. were considered). The total alignment contained 25 indels 1–385 bp in length (4 indels of 1–150 bp length in samples of *C*. *album* agg.). Seven cpDNA haplotypes were found in 122 analysed individuals of *C*. *album* agg. One haplotype was found in *C*. *ficifolium* (5 accessions analysed), two in *C*. *suecicum* (9 accessions), two in *C*. *strictum* (8 accessions) and one in *C*. *striatiforme* (2 accessions). The latter two species shared one haplotype (haplotype 1). The same haplotype was also found in hexaploid *C*. *album* s. str. In addition to this shared haplotype (haplotype 1), two further haplotypes were found within *C*. *album* s. str. (Fig 1).

Phylogenetic and haplotype network analyses revealed two major lineages of haplotypes, one diploid and one polyploid; the latter, however, did not receive significant bootstrap and posterior probability support (Fig 1). Whereas three haplotypes were found in diploid species (two of them formed a well-supported subclade in *C*. *suecicum*), 4 haplotypes were present in tetraploid and hexaploid species. No further subdivision correlated with species identity was found in the polyploid lineage.

### Analysis of ITS

#### Phylogenetic relationships and intraindividual polymorphism in *C*. *album* s. str

The total length of the ITS alignment was 625 bp (624 bp for *C*. *album* agg. taxa only), containing five 1 bp long indels (only one indel was found in *C*. *album* agg.). Forty-nine variable positions were found in *C*. *album* agg.

**Fig 2:**
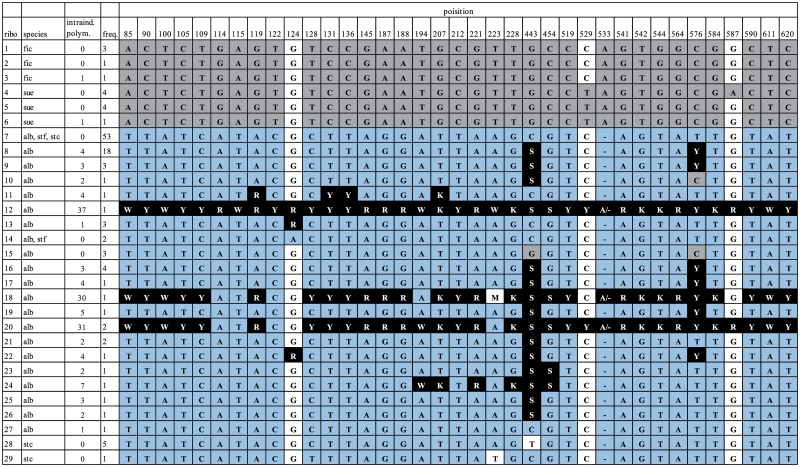
Nucleotide additivity in hexaploid *Chenopodium album* s. str. Columns represent the 37 sites discriminating between the diploid and tetraploid species. The nucleotide characters for all ribotypes are indicated. Sites where particular ribotypes showed intraindividual polymorphism are marked with IUPAC ambiguity codes (Y = C or T, M = C or A, W = A or T, K = T or G, R = A or G, S = C or G). For each ribotype (ribo), its presence in particular species (species) of the *C*. *album* group (fic = *C*. *ficifolium*, sue = *C*. *suecicum*, stc = *C*. *strictum*, stf = *C*. *striatiforme*, alb = *C*. *album* s. str.), the level of intraindividual polymorphism expressed as the number of additive sites (intraindividual polymorphism) and its frequency (freq) in the taxa analysed are given. Characters specific to diploid species are on a grey background. Characters specific to tretraploid species are on a blue background. Additive characters in hexaploid *C*. *album* s. str. are with white on a black background. Inconclusive characters are on a white background.

**Fig 3:**
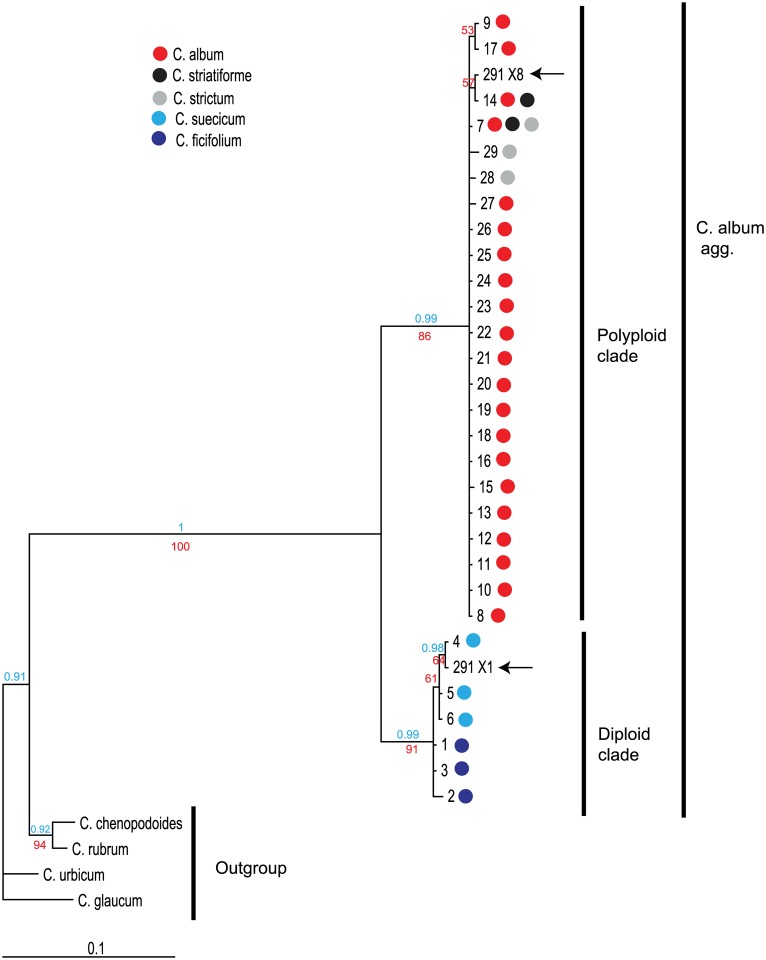
Phylogenetic analysis of the nrDNA ITS sequences. A 50% majority rule consensus tree of the Bayesian analysis is presented. Ribotypes are numbered according to Table 1 and Fig 2, and each ribotype is represented by one sequence. Numbers above and below branches indicate bootstrap support (red) and posterior probability (blue) values from the maximum parsimony and Bayesian analysis, respectively. The presence of the particular ribotypes in species of the *Chenopodium album* agg. is indicated by coloured dots (see legend). Sequences isolated from a hexaploid individual with a highly polymorphic direct sequence are indicated by black arrows.

Twenty-nine unique ITS sequences were revealed based on direct sequencing of 122 individuals of five *C*. *album* agg. species. In each of the diploid species, *C*. *ficifolium* and *C*. *suecicum*, three ribotypes were found. Two and three ribotypes were found in tetraploids *C*. *striatiforme* and *C*. *strictum*, respectively. While the sequences of the diploid and tetraploid species differed in 37 out of the 624 aligned characters (mean nucleotide diversity 1.3%), forming two well separated clades, the sequence divergence within the clades was small (mean nucleotide diversity 0.26% and 0.32% for diploids and polyploids, respectively) (Figs 2 and 3).

Three ribotypes of *C*. *suecicum* shared one synapomorphic mutation at position 529 (Fig 2) and formed a monophyletic group within the diploid clade (Fig 3). The sequences of *C*. *ficifolium* were placed to the base of this group.

Neither the sequences of *C*. *striatiforme* nor *C*. *strictum* formed a monophyletic group within the polyploid clade. Moreover, ribotype 7 was found in both species (Fig 3).

In *Chenopodium album* s. str., 21 ribotypes were identified, all of which were placed in the polyploid clade (Fig 3). The majority of these sequences (ribotypes 8–13, 16–27, see S1 Fig) were not homogenous and contained superimposed peaks at certain positions, indicating the presence of more than one sequence variant in these individuals. This intraindividual polymorphism varied largely between ribotypes (1–37 polymorphic sites), and most of the superimposed peaks occurred at positions where the sequences of diploids and tetraploids differed from each other and showed an additive pattern of diploid and tetraploid characters (Fig 2 and S1 Fig).

The majority of hexaploid individuals (54) possessed completely homogenized sequences, belonging to ribotypes 7, 14 and 15. The most frequent ribotype 7 was found in 50 hexaploid individuals. This ribotype, together with ribotype 14, was found also in tetraploids. Ribotype 15, found in 3 individuals, possesses tetraploid-like characters in all but two discriminative positions (443 and 576, Fig 2). At these sites, characters typical for diploid sequences were found, indicating that a chimeric sequence was maintained by concerted evolution.

Ribotypes with low levels of intraindividual polymorphism (containing 1–7 polymorphic sites) were also found. In these ribotypes (8–11, 13, 16, 17, 19, 21–27, see Fig 2 and S1 Fig), either mixed or tetraploid-like characters were found at positions discriminating between di- and tetraploids, indicating sequence homogenization towards tetraploid ribotypes.

**Fig 4:**
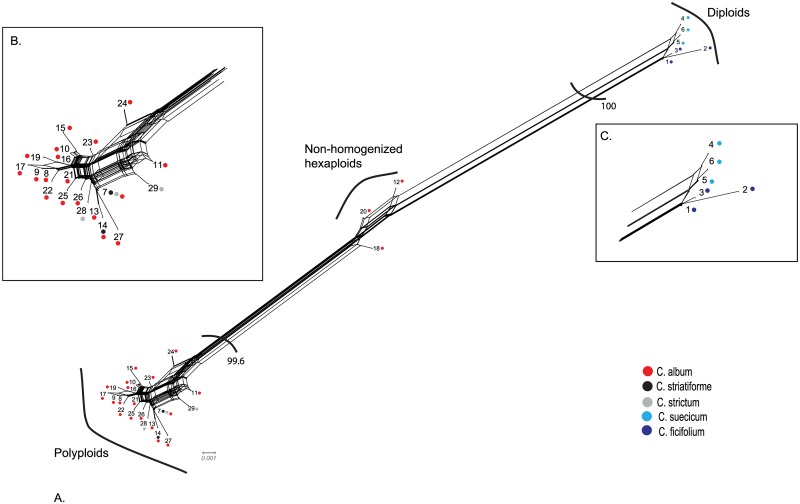
Neighbour net analysis of nrDNA ITS sequences. Ribotypes are numbered according to Table 1 and Fig 2, and each ribotype is represented by one sequence. The presence of particular ribotypes in different species of the *Chenopodium album* agg. is indicated by coloured dots (see legend). Relationships within the polyploid and diploid groups are depicted in detail (B, C, respectively).

We found only three ribotypes in four individuals that show complete (ribotype 12) or nearly complete (ribotypes 18 and 20) character state additivity in all 37 positions discriminating between the diploids and polyploids (Fig 2). Based on the results of neighbour net analysis, these sequences were placed at a position intermediate between the diploid and polyploid clades (Fig 4), illustrating the presence of an almost intact diploid-like ITS copy in addition to the tetraploid one in these individuals. The persistence of two different ITS copies in ribotype 12 was further confirmed by cloning and phylogenetic analyses. The cloned sequences showed 100% homology to ribotypes 4 and 14, characteristic of *C*. *suecicum* and *C*. *striatiforme*, respectively (Fig 3).

#### Geographic patterns of ITS sequence variation

We compared the composition and frequency of ribotypes both at the continental and local scales. The pattern of variation in ITS sequences did not reveal any geographic structure (S2 Fig). The most frequent ribotypes of all species were widely distributed across the sampled area. Some rare ribotypes were found hundreds or even thousands of kilometres apart; ribotype 15, for example, occurred in Central Europe, the Balkan Peninsula and the southwestern part of Siberia, the latter two locations being more than 4,000 km apart.

At local scale, the co-occurrence of individuals with different number of polymorphic positions was found in all five sampled populations (S3 Fig). The majority of sequences was completely or nearly completely homogenized towards the tetraploid parents. In all populations, the two most frequent ribotypes (ribotype 7 and 8) recognized at continental scale were also the most abundant ones. The exception is population 599, where eight ribotypes with more or less equal frequencies were found. In population 583, two individuals with ribotype 20 (representing completely unhomogenized sequences of both progenitors) were found.

### Genomic in situ hybridization

**Fig 5:**
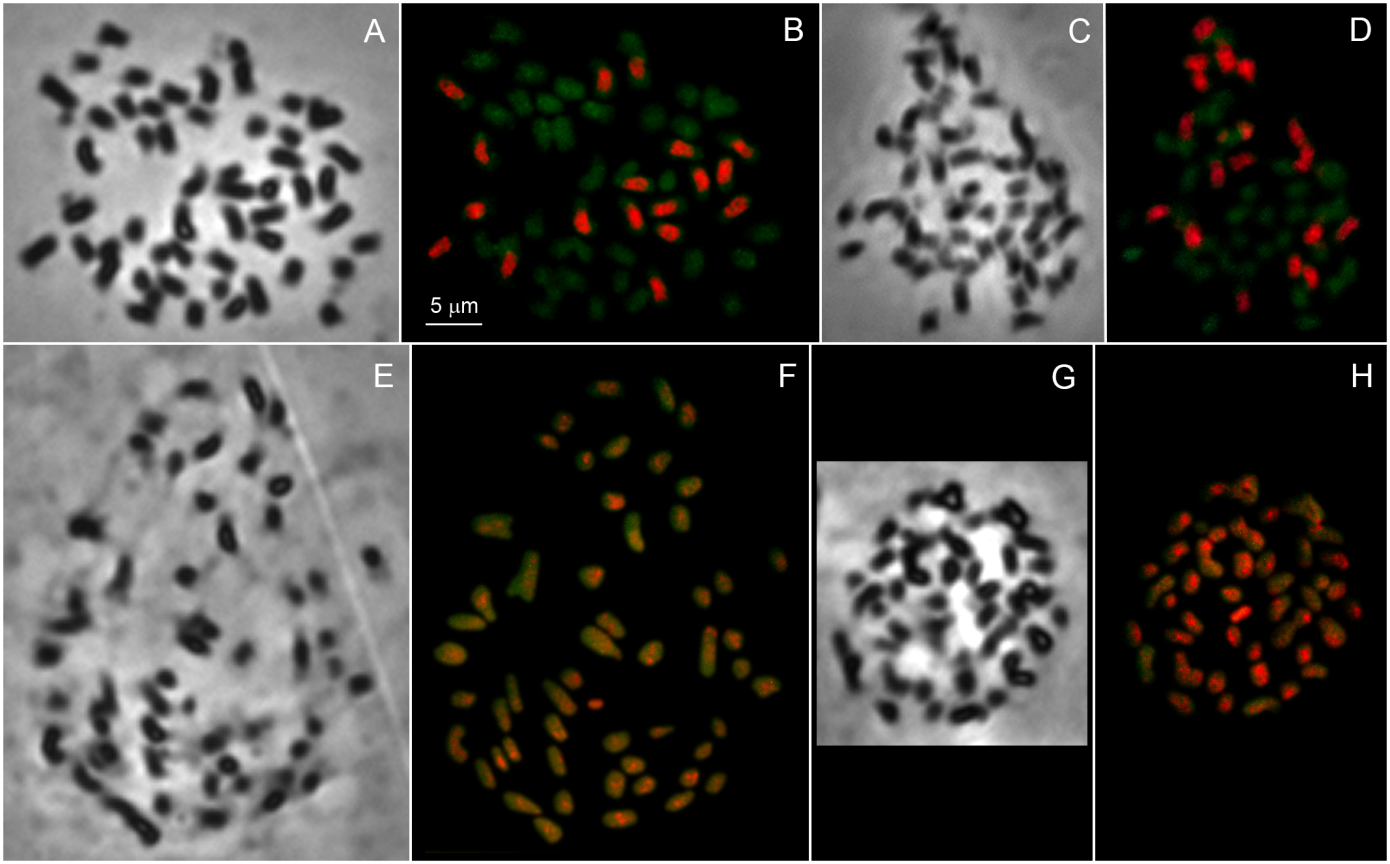
Genomic in situ hybridization on metaphase chromosomes of *Chenopodium album* s. str. (A,B) Phase-contrast image of *C*. *album* s. str. chromosomes (accession 457/10) and GISH on the same metaphase plate with a probe consisting of total DNA of *C*. *suecicum* (accession 277/10, orange fluorescence) plus total DNA of *C*. *strictum* (accession 380/1, green fluorescence). (C, D) Phase-contrast image of *C*. *album* s. str. chromosomes (accession 457/10) and GISH on the same metaphase plate with a probe consisting of total DNA of *C*. *ficifolium* (accession 276/10, orange fluorescence) plus total DNA of *C*. *strictum* (accession 380/1, green fluorescence). (E, F) Phase-contrast image of *C*. *album* s. str. chromosomes (accession 457/10) and GISH on the same metaphase plate with a probe consisting of total DNA of *C*. *strictum* (accession 380/1, orange fluorescence) plus total DNA of *C*. *album* s. str. (accession 329/1, green fluorescence). (G, H) Phase-contrast image of *C*. *album* s. str. chromosomes (accession 329/1) and GISH on the same metaphase plate with a probe consisting of total DNA of *C*. *striatiforme* (accession 331/5, orange fluorescence) plus total DNA of *C*. *album* s. str. (accession 329/1, green fluorescence).

Chromosomes of *C*. *album* were probed with total DNA of the putative diploid progenitors, *C*. *ficifolium* and *C*. *suecicum*, to determine the chromosomal distribution of common repetitive sequences. The intensity of red-orange fluorescence shows the level of affinity between repetitive sequences in the genomes tested. GISH reveal strong hybridization signal on 18 chromosomes of *C*. *album* with both diploid species *C*. *ficifolium* and *C*. *suecicum* (Fig 5B and 5D). These GISH experiments, using both diploid probes, resulted in the same intensity of fluorescent signal on the same chromosomes, confirming the close relatedness between the diploid taxa.

Probing chromosomes of *C*. *album* s. str. with the total DNA of related tetraploids *C*. *strictum* and *C*. *striatiforme* yielded ambiguous results. The hybridization mixture consisted of self-DNA of *C*. *album* (green fluorescence) and DNA of one of the proposed tetraploid ancestors (red fluorescence). A bright orange signal showed high homoeology of *C*. *strictum* and *C*. *striatiforme* DNA to all chromosomes of *C*. *album* s. str. both in euchromatic and in heterochromatic regions (Fig 5F and 5H), making it difficult to divide chromosomes into groups by the level of fluorescence.

Both, *C*. *album* s. str. with completely homogenized as well as non-homogenized ITS sequences were used for GISH and the results of these experiments did not differ.

## Discussion

### Allopolyploid origin of *Chenopodium album* s. str. and direction of hybridization

The origin of *C*. *album* s. str. has been a matter of discussions for several years. Different authors have proposed that this species may be of allopolyploid origin. However, controversy surrounds its progenitors [7, 37, 47, 48, 70].

Although we used several approaches, including the sequencing of ITS and cpDNA, and genomic in situ hybridization, none of them by itself has brought clear evidence concerning the origin of *C*. *album* s. str. However, all these approaches together have produced a collection of complementary evidence, suggesting that *C*. *album* s. str. originated by hybridization between a diploid and a tetraploid species, as suggested by Mandák et al. [7].

The GISH method with total genomic DNA as a probe provides us with unique information about similarities between repetitive DNA of related species as well as about the physical location of conserved sequences on chromosomes [71, 72]. The GISH results for diploid species, *C*. *ficifolium* and *C*. *suecicum*, show close relatedness to eighteen *C*. *album* s. str. chromosomes. The results indicate that the donor of these chromosomes was highly similar to present-day *C*. *ficifolium*/*C*. *suecicum*.

GISH experiments with tetraploid DNA as a probe resulted in fluorescent signal in eu- and heterochromatic chromosome regions (Fig 5F and 5H) on all 54 chromosomes of *C*. *album* s. str. This finding does not fit the hypothesis that *C*. *album* s. str. originated by hybridization between a diploid and a tetraploid progenitor. If this were the case, we would expect fluorescent signal only on the 36 tetraploid-derived chromosomes. However, this pattern could be explained by the presence of tribe-specific sequences [73], the presence of similar families of transposable elements, homogenization of intrapolyploid repetitive DNA sequences by concerted evolution [74] and interhaplome transfer at the tetraploid and hexaploid levels [72]. An additional investigation, including the analysis of repetitive sequence variation, is required to explain this pattern. However, as regards the allopolyploid origin of *C*. *album* s. str., the GISH method is especially valuable because it provides evidence about the involvement of diploid taxa.

The involvement of tetraploids is supported by cpDNA and ITS data. All hexaploid individuals analysed had a tetraploid-like cpDNA sequence. We therefore assume that tetraploids served as maternal parents in these crosses and that diploids were the pollen donors. ITS is a biparentaly inherited marker, so sequences of both parents can be anticipated in allopolyploids. However, most hexaploid accessions of *C*. *album* s. str. possessed only one sequence type, representing the tetraploid parent. The missing diploid sequences could be explained by their elimination due to concerted evolution [75], a series of intragenomic processes that may lead to complete homogenization of sequences across different loci [74]. We found some traces of diploid sequences in hexaploid individuals, representing partly homogenized sequences, as well as four individuals with a presence of intact diploid sequence. Similarly, intraspecific differences in the degree of sequence homogenization have also been reported for hybridogenous species of other genera (e.g. *Tragopogon* [76, 77], *Malus* [78], *Carapichea* [79]).

Mandák et al. [7] mention two possible mechanisms of allohexaploid *C*. *album* formation from diploid and tetraploid taxa: hybridization via fusion of unreduced gametes and a two-step procedure involving a triploid bridge. The fact that no triploids were found among the 482 plants analysed by Mandák et al. [7], not even in their enlarged dataset of 1977 accessions, covering most of the Eurasian distribution range of *C*. *album* agg. [46] speaks in favour of the first possibility. However, *C*. *album* s. str. forms dense and numerous populations, in which rare triploid individuals can be overlooked with high probability despite dense sampling. Another argument for the involvement of a triploid bridge in the evolution of *C*. *album* s. str. is that it could have played a role in the group’s evolutionary history even though no triploid has ever been found. On the other hand, the production of unreduced gametes in natural populations is relatively low (< 1%, see [80]). They, however, appear to be more frequent in populations exposed to environmental stress [81, 82], which could have increased the probability of fusion of two unreduced gametes and significantly accelerated the evolution of *C*. *album* s. str.

### Parental species remain unknown

Based on our data, no further conclusions can be drawn regarding the exact parental combination of *C*. *album* s. str. The GISH and genome size analysis of Mandák et al. [7] was unable to discriminate between species of the same ploidy level. Similarly, the analysis of cpDNA pointed towards the tetraploid as the maternal parent, but did not bring enough resolution to discriminate between the two tetraploid taxa. On the other hand, while *C*. *strictum* is a late-flowering tetraploid with no overlap in flowering time with diploid species, *C*. *striatiforme* is an early-flowering tetraploid and phenologically overlaps at least partly with the diploids. Flowering time might represent a pre-zygotic isolation mechanism favouring *C*. *striatiforme* as a putative donor of the tetraploid genome. Likewise, ITS allowed for only restricted discrimination between species of the same ploidy, due to a low level of sequence variation, a lack of phylogenetic structure and ribotype sharing between the two tetraploid taxa. This pattern is further complicated by a strong effect of concerted evolution in *C*. *album* s. str.

Walsh et al. [37] used a low-copy nuclear marker to elucidate the origin of polyploids within the *C*. *album* aggregate. They found *C*. *album* s. str. to be composed of three sub-genomes, but offered no conclusion as to their donors. It should be noted, however, that of all the putative parental taxa proposed by Mandák et al. [7], only *C*. *ficifolium* was included in the study of Walsh et al. [37]. The tetraploids *C*. *strictum* and *C*. *striatiforme* and the diploid *C*. *suecicum* were not sampled. The combination of comprehensive sampling and more variable markers (e.g., microsatellites and low-copy nuclear genes of Štorchová et al. [36] or Walsh et al. [37]), may be suitable for identifying the putative parents of *C*. *album* s. str.

### Polyphyletic origins of *C*. *album* s. str.

Recently, multiple origins, rather than a single one, have been confirmed for allopolyploid taxa from several genera such as *Arabidopsis* [16], *Arabis* [19], *Asplenium* [18] *Cardamine* [20] or *Tragopogon* [17, 21]. High morphological variation has repeatedly been described in *Chenopodium album* s. str. [22, 23, 44]. Mandák et al. [7] proposed that this variation could be explained by co-existence of multiple lineages of different origin within *C*. *album* s. str. We identified two ribotypes in *C*. *album* s. str. (ribotypes 7 and 14), which were completely homogenized and matched the sequences of putative parental taxa, *C*. *strictum* and *C*. *striatiforme*. We therefore assume that individuals of *C*. *album* s. str. possessing these two ribotypes have originated from different tetraploid genotypes (or even different taxa) and thus represent divergent genetic lineages of independent origin. The rest of the ribotypes found in the hexaploid accessions represent intermediate stages of still ongoing concerted evolution or recombinant sequences and therefore could not be used to estimate the number of genotypes within *C*. *album* s. str. Altogether, three cpDNA haplotypes were found in *C*. *album* s. str. The most abundant haplotype (haplotype 1) was also found in tetraploid species. The other two haplotypes were unique to *C*. *album* s. str. However, they are rare and closely related to haplotype 1. This pattern may indicate that there are more maternal lineages of *C*. *album* s. str. than revealed by ITS, but that they were not found among the tetraploids analysed due to insufficiently detailed sampling. On the other hand, the two haplotypes specific to *C*. *album* s. str. may have originated already after the formation of the hexaploid. As regards the supposed polyphyletic origin of *C*. *album* s. str., we can conclude that two independently formed lineages could be readily confirmed and that most likely this number is strongly underestimated.

### Ancient vs. recent hybridization

We found individuals with different degrees of ITS homogenization even within the same population. An extreme example is population 583, which besides individuals with partly or completely homogenized sequences comprised two non-homogenized accessions. The degree of sequence homogenization may be dependent on several factors. As reviewed in [75] besides the number and localization of rDNA loci, generation time and the time elapsed since the hybridization event may significantly affect the tempo of homogenization. Though there are numerous exceptions, in general it can be said that sequence homogenization should be more advanced in species with short generation times and in ancient hybrids [75]. All locations sampled in the Czech Republic for detailed analysis were in fact mixed populations of di-, tetra- and hexaploids. Such locations may represent ideal opportunities for natural hybridization. However, recent hybridization between taxa with different ploidy levels is considered to be rather rare [7, 83]. Mandák et al. [7] failed to experimentally synthetize hexaploid *C*. *album* s. str., suggesting that reproductive barriers between taxa with different ploidy level have already developed. However, hexaploid individuals with ITS sequences homogenized to different degrees may represent hybrids of different age. Those with unhomogenized sequences may be of recent origin whereas those with completely homogenized ones may be ancient. On the other hand, the rates of gene conversion are very specific, and it may be very misleading to estimate the time frame of a hybrid’s origin based on the patterns of sequence homogenization alone. Shortly after their formation, hybrids are known to exhibit additivity of their parental sequences [76, 84, 85], but one of the parental sequences may get eliminated from hybrids already after a few generations [85–87]. The most extreme example has been described for synthetic hybrids of *Armeria* [85], where one of the parental ribotypes was almost completely removed already in the F2 generation. By contrast, sequence homogenization in *Nicotiana* allopolyploids seems much slower, with complete elimination of one of the parental sequences taking hundreds of thousands to millions of years [88].

## Conclusions

Our study confirms that hexaploid *Chenopodium album* s. str. is an allopolyploid formed by hybridization between diploid and tetraploid taxa. All hexaploid accessions possessed cpDNA sequences identical or closely related to those of tetraploid taxa, indicating that tetraploids served as the maternal and diploid as the paternal parent. However, neither of the approaches used was able to identify the particular parental species for all hexaploid accessions, due to high sequence similarity of nrDNA ITS, cpDNA as well as repetitive sequences (as revealed by GISH) between species of the same ploidy level.

In hexaploid *C*. *album* s. str., nrDNA ITS sequences with different degrees of intraindividual polymorphism were identified. Most accessions were completely or nearly completely homogenized towards the maternal ribotype. A homogenous but apparently chimeric sequence, combining the characters specific to diploids and tetraploids, was found in three individuals. Both parental sequences were found only in four accessions. Variation in the nrDNA ITS region showed no apparent geographic pattern. Two separate nrDNA lineages, matching the variation within tetraploids, indicate that *C*. *album* s. str. originated multiple times. Hexaploid individuals with different degrees of sequence homogenization may represent allopolyploid lineages of different age.

## Supporting Information

S1 Fig Summary of variable sites in nrDNA ITS of *Chenopodium album* agg. Nucleotide characters of nrDNA ITS ribotypes identified in the present study in each of the 49 variable sites is indicated. Sites where particular ribotypes showed intraidividual polymorphism are marked by IUPAC ambiguity codes (Y = C or T, M = C or A, W = A or T, K = T or G, R = A or G, S = C or G). For each ribotype, the level of intraindividual polymorphism (as the “number of polymorphic sites”), its presence in particular species of the *C*. *album* group (fic = *C*. *ficifolium*, sue = *C*. *suecicum*, stc = *C*. *strictum*, stf = *C*. *striatiforme*, alb = *C*. *album* s. str.) and its frequency in the analysed taxa are given. Blue columns indicate sites discriminating between diploids and tetraploids or between individual species and that show character state additivity in hexaploids. Grey columns indicate sites that are additive in hexaploids; this additivity could be inferred from patterns of intraspecific variation of at least some of the putative parental species. Green columns indicate sites variable or polymorphic in some of the putative parental species but not additive in hexaploids. White columns indicate sites polymorphic in hexaploids but invariable in diploids and tetraploids.
(EPS)

S2 Fig Geographic distribution of nrDNA ITS ribotypes of *Chenopodium album* agg. (A) the continental scale and (B) in five populations from the Czech Republic.
(EPS)

S3 Fig Frequency of occurrence of nrDNA ITS ribotypes identified in hexaploid *Chenopodium album* s. str. at the continental wide and population level. Pie charts representing different populations from the Czech Republic are marked with population numbers according to Table 1. Charts named “all samples” indicate the frequency of ribotyopes in the complete dataset. (A) Frequency of occurrence of different ribotypes. (B) Frequency of occurrence of sequences with different numbers of intraindividual polymorphisms.
(EPS)
